# Serum Zinc-α2-Glycoprotein Levels Were Decreased in Patients With Premature Coronary Artery Disease

**DOI:** 10.3389/fendo.2019.00197

**Published:** 2019-03-29

**Authors:** Meijuan Liu, Huijuan Zhu, Tianshu Zhai, Hui Pan, Linjie Wang, Hongbo Yang, Kemin Yan, Yong Zeng, Fengying Gong

**Affiliations:** ^1^Key Laboratory of Endocrinology of National Health Commission, Department of Endocrinology, Peking Union Medical College Hospital, Chinese Academy of Medical Science and Peking Union Medical College, Beijing, China; ^2^Department of Cardiology, Beijing Anzhen Hospital, Capital Medical University, Beijing, China

**Keywords:** zinc-α2-glycoprotein (ZAG), premature coronary artery disease (PCAD), non-premature coronary artery disease (NPCAD), body mass index (BMI), diagnostic biomarker

## Abstract

**Objectives:** To explore serum zinc-α2-glycoprotein (ZAG) changes in patients with or without premature coronary artery disease (PCAD) and its association with several cardiovascular risk factors.

**Methods:** A total of 3,364 patients who were undergone coronary angiography in Peking Union Medical College Hospital were screened. According to the degree of coronary artery stenosis, the number of 364 patients with PCAD (age <55 years in males and <65 years in females) and 126 age and gender matched patients without premature coronary artery disease (NPCAD) were recruited in our present study. In addition, 182 age and gender matched healthy controls were also enrolled. Serum ZAG levels were determined by enzyme-linked immunosorbent assay (ELISA) method.

**Results:** Serum ZAG were significantly lower in the PCAD (8.03 ± 1.01 vs. 8.78 ± 1.89 μg/mL, *p* < 0.05) and NPCAD groups (8.28 ± 1.61 vs. 8.78 ± 1.89 μg/mL, *p* < 0.05), respectively, when compared with the controls. Multiple regression analysis showed that PCAD was independently associated with serum ZAG levels (*B* = −0.289, *p* = 0.002). The probability of PCAD in subjects with low tertile ZAG levels was 2.48-fold higher than those with high tertile levels after adjusting for other confounders [OR = 3.476, 95% CI 1.387–8.711, *p* = 0.008]. This phenomenon was more likely to be observed in male subjects with BMI <24 kg/m^2^. The receiver operating curve (ROC) analysis showed a weak diagnostic performance of serum ZAG for PCAD (AUC = 0.659, 95% CI 0.612–0.705, *p* < 0.05). At the cutoff value of 7.955 μg/mL serum ZAG, the sensitivity and specificity for differentiating patients with PCAD from controls were 50.5 and 78.0%, respectively. The combination of ZAG with other clinical variables including age, gender, BMI, SBP, FBG, TC, HDL-C, Cr, and Urea had significantly improved the diagnosis accuracy with a sensitivity of 82.6%, a specificity of 95.0%, and AUC of 0.957 (95% CI, 0.940–0.975, *p* < 0.05).

**Conclusion:** Serum ZAG levels were firstly found to be decreased in Chinese PCAD patients. Subjects with lower ZAG levels were more likely to have PCAD, especially for male subjects with BMI <24 kg/m^2^. ZAG might be the potential diagnostic biomarkers for PCAD patients, and the combination of ZAG and clinical variables had higher discriminative performance.

## Introduction

Coronary artery disease (CAD) is the leading cause of morbidity and mortality worldwide. According to the 2017 updated Heart Disease and Stroke statistics from the American Heart Association, 16.5 million persons aged ≥20 years in the United States have CAD (1). Furthermore, CAD accounts for about one-third of all deaths in adults aged over 35 years in the United States (1). In the past, studies about CAD usually concentrated on the old population. However, with the rapid development of the economy and the change of lifestyle, CAD is often encountered by young adults nowadays. It is estimated that approximately 4–10% of patients with documented CAD are <45 years (2, 3).

Premature coronary artery disease (PCAD), which is defined as the presence of any coronary artery stenosis ≥50% in males aged <55 years or females aged <65 years, has become more prevalent in recent years (4). Patients with PCAD need much more attention since its devastating effect on individuals, families and the society. However, it still remains a challenge to identify patients with PCAD early because the coronary angiography is the gold standard diagnostics for PCAD and it is much more complex and expensive (5). For this reason, there is necessary to identify novel and reliable biomarkers that could help to diagnose PCAD patients in its early phases.

PCAD is a complex multifactorial disease and its underlying mechanisms are still remaining unclear. Obesity, which means the excess adipose tissue has accumulated in the body, is a well-recognized strong risk factor for the occurrence and development of PCAD (6–8). White adipose tissue (WAT) is currently recognized as not only an energy storage organ but also as an active endocrine organ that secretes bioactive molecules called adipokines, such as adiponectin, leptin, tumor necrosis factor-α (TNF-α), and so on (9). A growing body of evidence indicates that several adipokines play crucial roles in PCAD process and may serve as the potential biomarkers of PCAD (9, 10). Omentin-1 and visfatin are such two kinds of adipokines which have been previously identified as potential serum biomarkers of PCAD with the receiver-operating characteristic (ROC) curve areas of 0.97 and 0.74, respectively, in Czechs population (11).

Zinc-α2-glycoprotein (ZAG, also called AZGP1) is a novel identified 43 kDa adipokine that has been demonstrated to play an important role in the regulation of body weight, glucose, and lipid metabolism (12, 13). Moreover, circulating ZAG levels are closely associated with traditional cardiovascular risk factors, such as obesity (14–16), diabetes (17), hypertension (18), cigarette smoking (19), and so on. Thus, it is reasonable for us to wonder whether ZAG has any effect on PCAD development and progression. Previous studies by Smékal et al. in 65 Caucasian PCAD patients have demonstrated that serum ZAG levels in patients were decreased when compared with the controls (11). Further analysis showed that the area under the curve (AUC) of the ROC curve of ZAG was 0.89, which suggested that ZAG might be used as a potential serum biomarker for the diagnosis of PCAD patients (11). However, it is still unclear whether serum ZAG levels are changed in Chinese PCAD patients and whether it can be used as a biomarker for the diagnosis of PCAD patients in Chinese population.

Therefore, in this study, we aimed to investigate, for the first time, the association between serum ZAG levels and PCAD in Chinese Han population. Additionally, the potential usefulness of circulating ZAG as non-invasive diagnostic biomarkers for PCAD was also evaluated.

## Materials and Methods

### Study Subjects

A clinical medical database which includes a total of 3,364 subjects who had undergone coronary angiography in Peking Union Medical College Hospital (PUMCH) from November 2011 through April 2016 has been established in our previous study. We screened the database and selected the PCAD patients and NPCAD patients (without PCAD) as the following inclusion and exclusion criteria. The patients in PCAD group were any coronary artery stenosis ≥50% with age male <55 years or female <65 years (20, 21). The patients in NPCAD group were all coronary artery stenosis <50% and their age, sex, and BMI were matched with the patients in PCAD group. Patients who already had coronary artery stent or bypass graft or those were found myocardial bridge during coronary angiography were excluded in the study. Besides, patients with the infectious diseases, autoimmune diseases, renal or hepatic diseases, aortic dissection, and aneurysm were also excluded. A total of 490 subjects were finally enrolled in the study, which consisted of 364 participants with PCAD (PCAD group) and 126 subjects without PCAD (NPCAD group). In addition, 182 subjects (age, sex, and BMI matched) who had physical examination at the Medical Center in PUMCH from 2009 to 2012 and had normal liver, kidney, routine blood and urine tests, systolic blood pressure (SBP) and diastolic blood pressure (DBP) were collected as controls. The study was approved by the ethics committee of PUMCH (No. S-K205) and all participants signed written informed consent.

### Blood Sample Collection and Laboratory Analysis

Venous blood samples were taken from all participants after an overnight fast before angiography. Serum total cholesterol (TC), triglycerides (TG), low-density lipoprotein cholesterol (LDL-C), high-density lipoprotein cholesterol (HDL-C), fasting blood glucose (FBG) levels, liver and kidney function were determined by routine automated laboratory methods in our clinical laboratory. Serum ZAG levels were measured by a commercially available enzyme-linked immunosorbent assay (ELISA) kit (USCN Life Science Inc. Wuhan, China) according to the manufacturer's instructions. The minimum detectable concentration of ZAG was 1.80 ng/mL. The intra- and inter-assay coefficients of variation were 3.08 and 15.32%.

### Coronary Angiography

After preoperative preparation, the angiography was performed by the experienced interventional cardiologist using a quantitative coronary angiographic system. CAD was defined by standard criteria (the American College of Cardiology/American Heart Association guidelines) as the presence of ≥50% stenosis of the lumen diameter in at least one major coronary artery (22).

### Statistical Analyses

Data were shown as the mean ± standard deviation (SD). Normal distribution of the data was evaluated using the Shapiro-Wilk test. Comparisons for normally distributed continuous data were performed by either the independent sample *t*-test or one-way ANOVA analysis, as appropriate. Non-normally distributed variables were natural log (ln)-transformed before the analyses. Multiple linear regression was employed to determine variables that had independent associations with serum ZAG. Univariate and multivariate logistic regression analysis were used to estimate the odds ratio (OR) and 95% confidence intervals (CI) of serum ZAG levels for PCAD. Cut-off point analysis which defined by the largest distance from the diagonal line of the ROC [sensitivity × (1-specificity)] was used to identify the optimal value of serum ZAG levels which could differentiates PCAD patients from controls. The sensitivity and specificity of the index for the cut-off point were also calculated by using R version 3.3.4 (Foundation for Statistical Computing, Vienna, Austria) equipped with the “qROC” packages. All statistical analyses were performed with SPSS version 20.0 for Windows (SPSS Inc., Chicago, IL, USA). *p* < 0.05 was considered as statistically significant.

## Results

### General Characteristics of Subjects in PCAD, NPCAD, and Control Groups

Demographic, clinical and biochemical characteristics of PCAD and NPCAD patients as well as controls are shown in Table 1. When considering cardiovascular risk factors, SBP, DBP, FBG, TC, and TG were significantly higher, while HDL-C was lower in PCAD group as compared with the age, sex, BMI matched controls (all *p* < 0.05). Although patients in NPCAD group cannot be diagnosed as PCAD due to all coronary artery stenosis <50%, the cardiovascular risk factors including SBP, FBG, and TG were also significantly higher, and HDL-C was lower in NPCAD group in comparison with controls (all *p* < 0.05). In addition, Urea and Cr were significantly higher both in PCAD and NPCAD groups, and ALT was significantly higher only in PCAD but not in NPCAD, when compared with controls (all *p* < 0.05). Moreover, FBG and LDL-C were significantly higher while HDL-C was lower in PCAD patients than that in NPCAD patients. However, TG and Urea were much lower in PCAD patients in comparison with that in NPCAD patients, which may be related to the use of the drugs in PCAD patients.

**Table 1:** The demographic, clinical and biochemical characteristics of the study participants.

**Characteristics**	**Control group (*n* = 182)**	**NPCAD group (*n* = 126)**	**PCAD group (*n* = 364)**
Age (years)	51.22 ± 9.24	52.10 ± 7.47	52.34 ± 6.16
Sex (M/F)	109/73	61/65	182/182
BMI (kg/m^2^)	25.46 ± 3.18	26.13 ± 3.10	25.49 ± 3.39
SBP (mmHg)	116.82 ± 11.36	125.98 ± 14.70^a^	126.98 ± 17.69^a^
DBP (mmHg)	74.21 ± 7.42	74.96 ± 12.14	76.70 ± 11.74^a^
FBG (mmol/L)^#^	5.16 ± 0.66	6.22 ± 2.17^a^	7.81 ± 3.49^a^^b^
TC (mmol/L)^#^	4.91 ± 0.91	9.14 ± 12.73	4.94 ± 5.04^a^
TG (mmol/L)^#^	1.61 ± 1.60	2.64 ± 1.89^a^	2.11 ± 2.09^a^^b^
HDL-C (mmol/L)^#^	1.34 ± 0.30	1.25 ± 0.77^a^	1.04 ± 0.52^a^^b^
LDL-C (mmol/L)	3.08 ± 0.72	2.29 ± 1.00^a^	2.63 ± 0.96^a^^b^
ALT (U/L)^#^	25.79 ± 16.53	26.04 ± 17.27	41.27 ± 57.49^a^
Cr (μmol/L)^#^	66.36 ± 22.61	71.55 ± 16.18^a^	72.37 ± 22.84^a^
Urea (mmol/L)^#^	5.23 ± 1.30	21.33 ± 27.62^a^	6.93 ± 9.62^a^^b^
Statins (%)	0	32.41	34.54
Oral antidiabetic drugs (%)	0	13/107 (12.15)	75/305 (24.59)
Insulin (%)	0	6/105 (5.71)	22/292 (7.53)
ZAG (μg/mL)	8.78 ± 1.89	8.28 ± 1.61^a^	8.03 ± 1.01^a^
Male	8.94 ± 2.06	8.49 ± 1.88	8.11 ± 1.38^a^
Female	8.55 ± 1.60	8.08 ± 1.30^a^	7.94 ± 1.23^a^

*Values are expressed as the mean ± SD*.

*PCAD, premature coronary artery disease; NPCAD, non-premature coronary artery disease; BMI, body mass index; SBP, systolic blood pressure; DBP, diastolic blood pressure; FBG, fasting blood glucose; TC, total cholesterol; TG, triglycerides; HDL-C, high-density lipoprotein cholesterol; LDL-C, low-density lipoprotein cholesterol; ALT, alanine aminotransferase; Cr, creatinine; Urea, uricemia; ZAG, zinc-α2-glycoprotein*.

# *Variables were ln-transformed before analysis*.

a *p < 0.05 compared with control group*.

b *p < 0.05 compared with NPCAD group*.

### Serum ZAG Levels in PCAD, NPCAD Patients, and Controls

**Figure 1:**
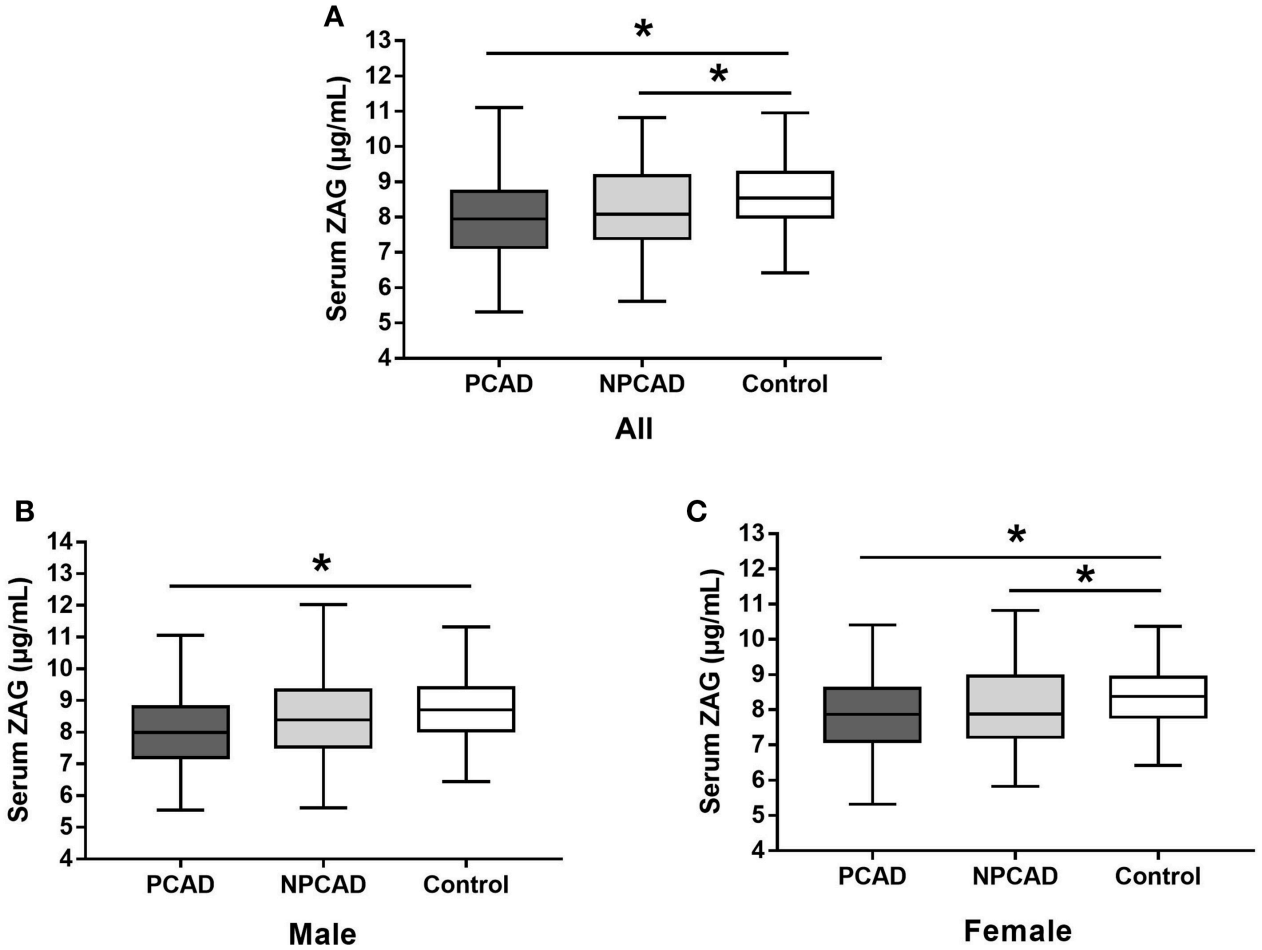
Serum ZAG levels of all subjects **(A)**, male **(B)**, and female **(C)** in PCAD, NPCAD patients, and controls. ZAG, zinc-α2-glycoprotein; PCAD, premature coronary artery disease; NPCAD, non-premature coronary artery disease. All values are expressed as the mean ± SD. ^*^p < 0.05.

As shown in Figure 1A, serum ZAG levels were significantly lower in PCAD patients in comparison with the controls (8.03 ± 1.01 vs. 8.78 ± 1.89 μg/mL, *p* < 0.05). This phenomenon was still remained when this comparison was performed, respectively, in males (8.11 ± 1.38 vs. 8.93 ± 2.06 μg/mL, *p* < 0.05) and females (7.94 ± 1.23 vs. 8.55 ± 1.60 μg/mL, *p* < 0.05) (Figures 1B,C). Moreover, serum ZAG levels in NPCAD patients were also significantly decreased (8.28 ± 1.61 vs. 8.78 ± 1.89 μg/mL, *p* < 0.05) when compared with the controls (Figure 1A). However, the decreased serum ZAG levels in NPCAD patients was only remained in the female, but not in male (Figures 1B,C). In addition, no significant difference was observed in serum ZAG levels between PCAD and NPCAD patients.

Next, given a significant proportion of patients in PCAD/NPCAD group in our present study are diabetic, serum ZAG levels in PCAD, and NPCAD groups were further analyzed when diabetic patients were removed. As shown in Figure S1A, serum ZAG levels were significantly lower in PCAD patients when compared with the controls (8.10 ± 1.30 vs. 8.78 ± 1.89 μg/mL, *p* < 0.05) like what we have observed in all subjects. This phenomenon was still remained when this comparison was performed, respectively, in males (8.18 ± 1.43 vs. 8.94 ± 2.06 μg/mL, *p* < 0.05) and females (8.02 ± 1.15 vs. 8.55 ± 1.60 μg/mL, *p* < 0.05) (Figures S1B,C). Moreover, serum ZAG levels in NPCAD patients were also significantly decreased (8.40 ± 1.70 vs. 8.78 ± 1.89 μg/mL, *p* < 0.05) when compared with the controls like what we have observed in all subjects (Figure S1A). No significant difference was observed in serum ZAG levels between PCAD and NPCAD patients.

### Associations of Serum ZAG Levels and Clinical Parameters in All Subjects

As displayed in Table 2, multivariate linear regression analysis showed that group, age, gender, BMI, and Cr were independent factors associated with serum ZAG levels. Among them, the presence of PCAD was found to be negatively associated with serum ZAG levels (β = −0.170, *p* = 0.002), which was consistent with the lower serum ZAG levels in PCAD patients as displayed in Figure 1A. In addition, serum ZAG levels were negatively related to gender (β = −0.142, *p* = 0.018) and BMI (β = −0.090, *p* = 0.046), but positively related to age (β = 0.167, *p* = 0.004) and Cr (β = 0.098, *p* = 0.040).

**Table 2:** Multiple regression analysis of variables independently related with serum ZAG in all subjects.

**ZAG (*R*^**2**^ = 0.075)**	**Linear regression analysis**		
	**Unstandardized coefficients (B) (95% CI)**	**Standardized coefficients (β)**	***p***
Constant	6.446 (4.565 to 8.327)		**0.000**
Group (Control, NPCAD, PCAD)	−0.289 (−0.471 to 0.108)	−0.170	**0.002**
Age	0.031 (0.010 to 0.053)	0.167	**0.004**
Gender (Male, Female)	−0.392 (−0.716 to 0.068)	−0.142	**0.018**
BMI (< 24, ≥24)	−0.255 (−0.506 to 0.005)	−0.090	**0.046**
SBP	0.003 (−0.005 to 0.010)	0.033	0.467
FBG^#^	−0.194 (−0.577 to 0.189)	−0.049	0.319
TC^#^	0.103 (−0.186 to 0.392)	0.038	0.486
HDL-C^#^	−0.139 (−0.614 to 0.337)	−0.030	0.567
Cr^#^	0.329 (0.014 to 0.644)	0.098	**0.040**
Urea^#^	0.154 (−0.108 to 0.416)	0.061	0.248

*Bold p-values indicate statistical significance (p < 0.05)*.

# *Variables were ln-transformed before analysis*.

*ZAG, zinc-α2-glycoprotein; CI, confidence intervals; NPCAD, non-premature coronary artery disease; PCAD, premature coronary artery disease; BMI, body mass index; SBP, systolic blood pressure; FBG, fasting blood glucose; TC, total cholesterol; HDL-C, high-density lipoprotein cholesterol; Cr, creatinine; Urea, uricemia*.

### Association of Serum ZAG Tertiles With PCAD/NPCAD Risks

In order to further investigate the relationship of serum ZAG levels and PCAD risks, the unconditional logistic regression analysis was performed, and subjects in PCAD and control groups were stratified into trisections according to ZAG tertiles (lowest: < 7.711 μg/mL; median: 7.711–8.637 μg/mL; highest: ≥8.637 μg/mL). As presented in Table 3, the probability of the PCAD in patients with the low ZAG levels was 1.765-fold higher than those with the high ZAG levels after adjusting for age, gender, BMI, SBP, and ln-FBG (Model 1, OR = 2.765, 95% CI 1.332–5.742, *p* = 0.006). This increased probability of PCAD risk still remained after further adjusting for ln-TC and ln-HDL-C based on Model 1 (Model 2, OR = 2.977, 95% CI 1.259–7.037, *p* = 0.013) and ln-Cr and ln-Urea based on Model 2 (Model 3, OR = 3.476, 95% CI 1.387–8.711, *p* = 0.008). Given the close relationship between serum ZAG levels and diabetes, the unconditional logistic regression analysis without diabetic patients were also performed. As displayed in Table S1, when diabetic patients were removed, the probability of the PCAD in patients with the low ZAG levels was 1.657-fold higher than those with the high ZAG levels after adjusting for age, gender, BMI, SBP, and ln-FBG (Model 1, OR = 2.657, 95% CI 1.261–5.595, *p* = 0.010). This increased probability of PCAD risk still remained after further adjusting for ln-TC and ln-HDL-C based on Model 1 (Model 2, OR = 3.006, 95% CI 1.246–7.253, *p* = 0.014) and ln-Cr and ln-Urea based on Model 2 (Model 3, OR = 3.648, 95% CI 1.432–9.292, *p* = 0.007).

**Table 3:** Unconditional logistic regression analysis of PCAD/NPCAD risks according to the tertiles of serum ZAG.

**Measurement**	**Tertile**		
	**Lowest OR (95% CI)**	**Median OR (95% CI)**	**Highest OR (95% CI)**
**ZAG in PCAD vs. Control**			
Range (μg/mL)	< 7.711	≧7.711 to < 8.637	≧8.637
Cases/controls	151/31	114/68	99/83
Model 1	2.765 (1.332–5.742)	1.487 (0.756–2.923)	1.00 (reference)
*p*	**0.006**	0.250	
Model 2	2.977 (1.259–7.037)	1.438 (0.637–3.244)	1.00 (reference)
*p*	**0.013**	0.382	
Model 3	3.476 (1.387–8.711)	2.013 (0.830–4.883)	1.00 (reference)
*p*	**0.008**	0.122	
**ZAG in NPCAD vs. Control**			
Range (μg/mL)	< 7.980	≧7.980 to < 8.925	≧8.925
Cases/controls	60/42	26/77	40/63
Model 1	2.916 (1.332–6.383)	1.00 (reference)	2.666 (1.168–6.086)
*p*	**0.007**		**0.020**
Model 2	3.044 (1.151–8.049)	1.00 (reference)	2.638 (0.999–6.963)
*p*	**0.025**		0.050
Model 3	3.291 (1.135–9.548)	1.00 (reference)	2.433 (0.828–7.151)
*p*	**0.028**		0.106
**ZAG in PCAD vs. NPCAD**			
Range (μg/mL)	< 7.479	≧7.479 to < 8.520	≧8.520
Cases/controls	125/38	123/41	116/47
Model 1	1.417 (0.821–2.447)	1.430 (0.840–2.436)	1.00 (reference)
*p*	0.211	0.188	
Model 2	1.152 (0.629–2.109)	1.164 (0.637–2.126)	1.00 (reference)
*p*	0.647	0.622	
Model 3	1.172 (0.628–2.188)	1.071 (0.576–1.991)	1.00 (reference)
*p*	0.619	0.830	

*Multivariate odds ratios (OR) and 95% confident intervals (CI) from unconditional logistic regression models were used in the analysis. Bold p-values indicate statistical significance (p < 0.05)*.

*Model 1: basic model, adjusted for age, gender, BMI (< 24 kg/m^2^, ≥24 kg/m^2^), SBP and ln-FBG*.

*Model 2: further adjusted for ln-TC and ln-HDL-C based on the model 1*.

*Model 3: Full model, further adjusted for ln-Cr and ln-Urea based on the model 2*.

Serum ZAG levels in NPCAD and control groups were also stratified into trisections (lowest: < 7.980 μg/mL; median: 7.980–8.925 μg/mL; highest: ≥8.925 μg/mL). As showed in Table 3, both low and high levels of ZAG showed a significant increased risk of NPCAD when compared with the reference group of the middle ZAG levels after adjusting for age, gender, BMI, SBP, and ln-FBG (Model 1, OR = 2.916, 95% CI 1.332–6.383, *p* = 0.007; OR = 2.666, 95% CI 1.168–6.086, *p* = 0.020, respectively). However, when further adjustment ln-TC and ln-HDL-C in Model 2, and even further adjustment for ln-Cr and ln-Urea in Model 3, the increased risk of NPCAD was only observed in patients with the low ZAG levels (Model 2, OR = 3.044, 95% CI 1.151–8.049, *p* = 0.025; Model 3, OR = 3.291, 95% CI 1.135–9.548, *p* = 0.028, respectively), but not in patients with the high ZAG levels. Additionally, in PCAD and NPCAD groups, the changes of serum ZAG levels were not associated with the risk of PCAD (Table 3). When diabetic patients in PCAD and NPCAD groups were removed, serum ZAG levels were also not associated with the probability of PCAD (Table S1).

**Figure 2:**
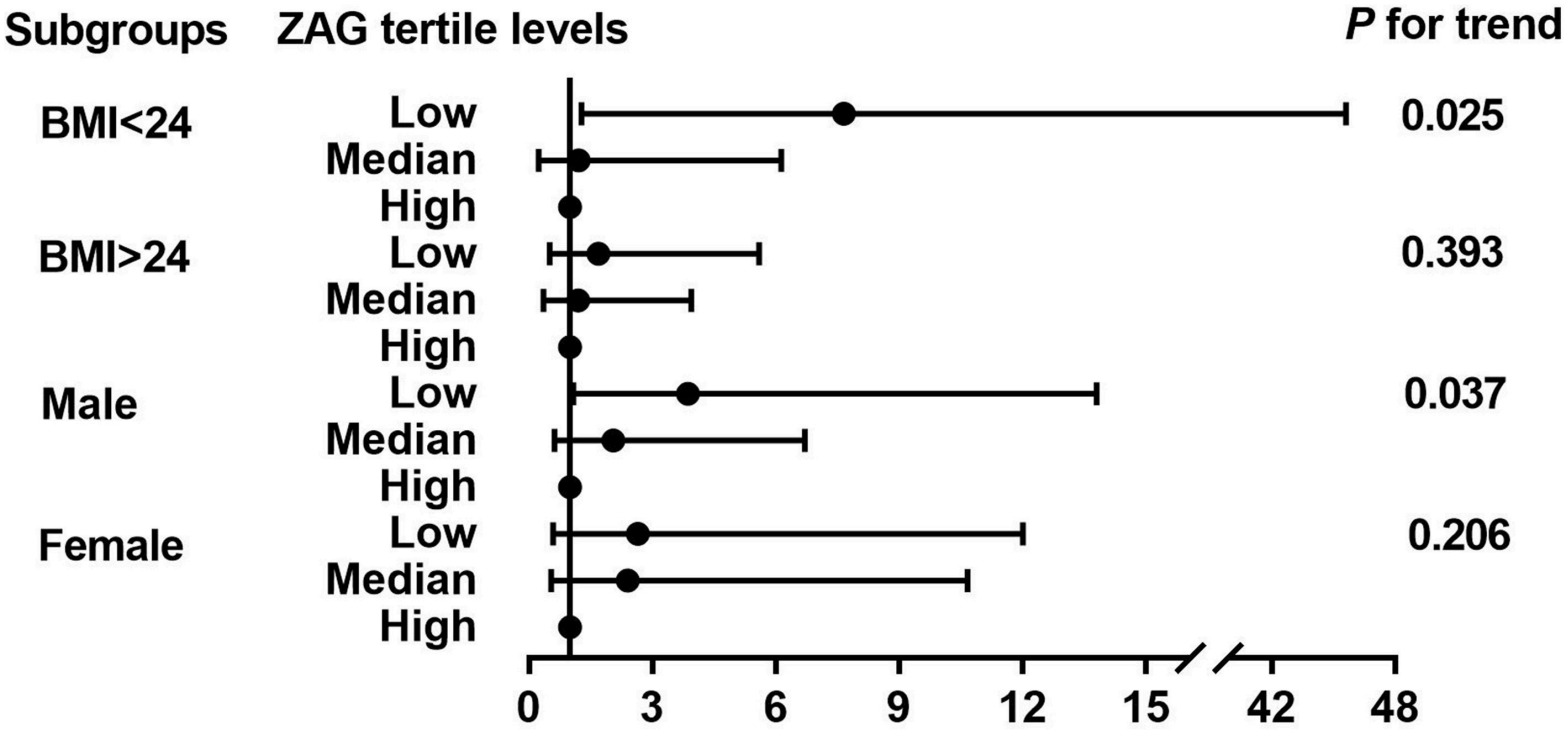
Further logistic regression analysis of PCAD risks according to tertiles of ZAG in subgroups analyses. Multivariate odds ratios (OR) and 95% confident interval (CI) from unconditional logistic regression models were used in the analysis, adjusted for age, gender (male, female), BMI (<24 kg/m^2^, ≥24 kg/m^2^), SBP, FBG, TC, HDL-C, Cr, Urea. Stratified variables were also adjusted for in the subgroup analysis when possible.

Next, the detailed unconditional logistic regression subgroup analysis of the association between the tertile ZAG levels and PCAD risks was further conducted after adjusting for age, gender, BMI, SBP, ln-FBG, ln-TC, ln-HDL-C, ln-Cr, and ln-Urea in PCAD patients and controls. As demonstrated in Figure 2, subjects with the low ZAG levels in our present study were more likely to have PCAD than those with the high ZAG levels, especially in males with BMI <24 kg/m^2^.

### Diagnostic Values of Serum ZAG for PCAD

**Figure 3:**
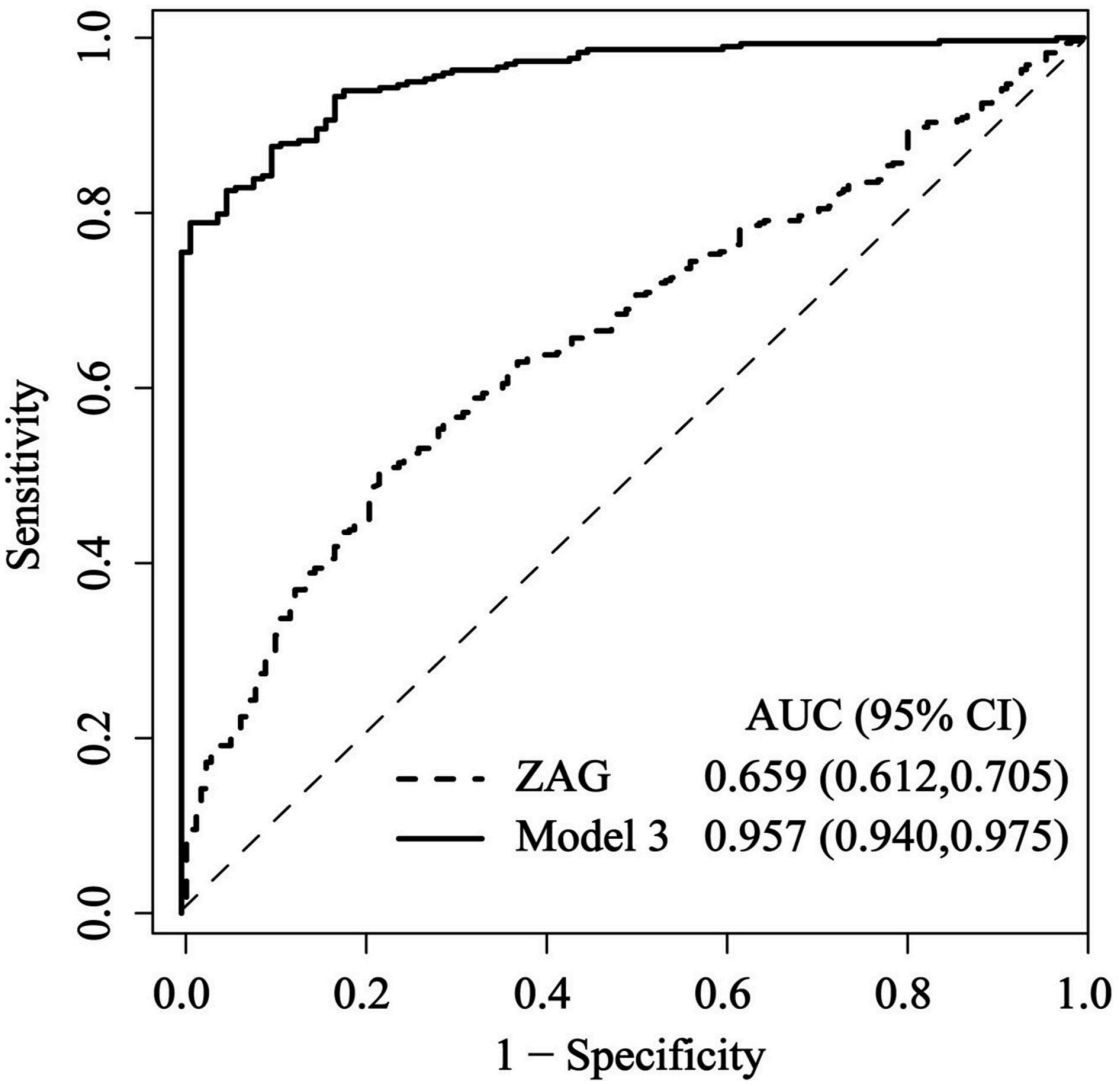
Comparison for ROC curves of serum ZAG alone and the combination of ZAG with other clinical variables (in Model 3 of Table 3) in PCAD patients and controls. ROC curves were derived by plotting the relationship between the specificity and the sensitivity at various cutoff levels. ZAG, zinc-α2-glycoprotein; ROC, receiver operating characteristic; AUC, area under the curve.

Finally, to investigate the predictive value of ZAG for PCAD, ROC curve analysis was performed by using R version 3.3.4 equipped with the “qROC” packages. As shown in Figure 3, ZAG could be a potential diagnostic biomarker for differentiating PCAD patients from controls. The optimal ZAG concentration that was used as the cutoff value was 7.955 μg/mL with a sensitivity of 50.5%, a specificity of 78.0%, and AUC of 0.659 (95% CI 0.612–0.705, *p* < 0.05). Furthermore, when both ZAG and other clinical variables including age, gender, BMI, SBP, FBG, TC, HDL-C, Cr, and Urea (in Model 3 of Table 3) were taken into the analysis, the ROC curve area (AUC) was increased to 0.957 (95% CI, 0.940–0.975, *p* < 0.05) with a sensitivity of 82.6%, a specificity of 95.0% as presented in Figure 3. When diabetic patients in PCAD and NPCAD groups were removed, ZAG also showed a diagnostic performance for PCAD (AUC = 0.617, 95% CI 0.569–0.666, *p* < 0.05), and the combination of ZAG and clinical variables had higher discriminative performance (AUC = 0.829, 95% CI 0.786–0.871, *p* < 0.05) (Figure S2).

## Discussion

CAD, accounting for 14.8% of global death, is still a worldwide public health burden (23). Premature coronary artery disease (PCAD) is defined as the presence of any coronary artery stenosis ≥50% in males aged <55 years or females aged <65 years (4). With the prevalence of the adverse lifestyles, such as smoking, drinking, high-fat diets, physical inactivity, and so on, even in children and young adults, the proportion of PCAD is continually growing (24), and PCAD contributes to about 30% of all CAD subjects (2, 25). More seriously, PCAD generates much more devastating effects on individuals, families and the society (26). Thus, the identification and diagnosis of PCAD as early as possible is quite important. ZAG is a newly described 41-kDa adipocytokine that can be secreted by adipocytes (13). Our previous studies together with others have revealed the close association between serum ZAG levels and traditional cardiovascular risk factors for PCAD, including obesity (14–16), type 2 diabetes mellitus (27), hypertension (18), dyslipidemia (12, 28), chronic kidney disease (29, 30), polycystic ovary syndrome (31), and smoking (19). Studies performed by Qu et al. (27) and Tsai et al. (19) indicated that serum ZAG might serve as a new biomarker for identifying insulin resistance and metabolic syndrome. In our present study, we firstly found that serum ZAG levels were significantly lower in Chinese PCAD patients in comparison to the controls. This phenomenon was still remained even when diabetic patients in PCAD group were removed. Our observation of lower serum ZAG levels in PCAD patients is supported by the study performed by Smékal et al. who also demonstrated the significantly lower serum ZAG levels in PCAD patients (*n* = 65) when compared with controls (*n* = 50) in Czechs population (11). Interestingly, the present study also firstly found that PCAD was independently associated with serum ZAG levels after adjusting for age, gender and other anthropometric and biochemical items. After stratifying subjects in PCAD and control groups into trisections according to their serum ZAG tertiles, the probability of PCAD in subjects with the low ZAG levels was 1.77-fold higher than those with the high levels after adjusting for age, gender, BMI, SBP, and ln-FBG (Model 1). This increased probability of PCAD risk in subjects with low ZAG levels still remained after further adjusting for ln-TC and ln-HDL-C based on Model 1 (Model 2) and ln-Cr and ln-Urea based on Model 2 (Model 3), especially for male subjects with BMI <24 kg/m^2^. It is noteworthy that even in NPCAD patients, whose all coronary artery stenosis <50%, individuals with the low ZAG levels had 2.29-fold higher risks of NPCAD than those in the reference group of median ZAG levels even after full adjustment for NPCAD risk factors. Although patients with NPCAD cannot be diagnosed as PCAD according to the results of coronary angiography, the abnormalities in glycolipid and cardiovascular metabolism have already emerged. This result indicated that the reduction of serum ZAG levels was not only a risk factor for PCAD, but also as a risk factor for NPCAD with abnormal glycolipid and cardiovascular metabolism. In support of our findings, previous studies have shown the negative association between serum ZAG and cardiovascular risk factors. Leal et al. demonstrated that serum ZAG was inversely associated with markers of pro-atherogenic factors, such as TNF-α and vascular cell adhesion molecule 1 (VCAM-1) in hemodialysis patients (32). Further studies conducted by Sörensen-Zender et al. in experimental mice showed that the deletion of ZAG exacerbated experimental mice cardiac fibrosis, which also indicated the negative regulation role of ZAG in cardiovascular disease development (33). It is thus reasonable to hypothesize that lower circulating levels of ZAG observed in our study may participate in the development and progression of PCAD, and ZAG may be a novel marker of the diagnosis of PCAD.

Smékal et al. reported that serum ZAG was a useful diagnostic marker for PCAD in a cohort of 190 Czechns individuals (11). They found that at the cutoff value of 51.7 mg/L (ELISA kit, Max002 Dynatech, Biovendor, Brno, Czech Republic), the AUC of ZAG was 0.894 (95% CI 0.785–0.913) with 73.3% sensitivity and 86.6% specificity (11). In our present study, we also found that at the cutoff value of 7.955 μg/mL (ELISA kit, USCN Life Science Inc. Wuhan, China), serum ZAG could discriminate PCAD patients from controls with ROC curve area of 0.659 and 50.5% sensitivity and 78.0% specificity, respectively. Our further analysis showed that a combination of ZAG and clinical variables (BMI, SBP, FBG, TC etc. in Model 3 of Table 3) produced much better discriminative performance than ZAG alone with ROC curve areas of 0.957 (95% CI, 0.940–0.975), and 82.6% sensitivity and 95.0% specificity. The diagnostic value of ZAG was still remained when diabetic patients in PCAD and NPCAD groups were removed. Our present findings together with others suggest that ZAG could be used as a potential serum biomarker for PCAD, and the combination of ZAG and clinical variables could yield a better discriminatory power.

It is also of interest in our current study to note that BMI was negatively associated with serum ZAG levels in PCAD patients, even after adjusting for the general clinical and laboratory parameters. Consistent with our findings in PCAD patients, our previous studies in overweight/obese subjects as well as in high fat diet (HFD) -induced obese mice also found an inverse correlation between serum ZAG and body weight and fat mass (all *P* < 0.05) (14, 16). In support of our results, previous studies by others also found the negative correlation between circulating ZAG and BMI in type 2 diabetes mellitus (34) and in patients with newly diagnosed metabolic syndrome (35). Furthermore, the negative relationship between ZAG and obesity was further verified in ZAG administration and ZAG knockout mice, which showed that ZAG administration could induce a rapid and significant reduction in body weight in *ob/ob* and HFD-induced obese mice (36, 37), while ZAG knock-out mice gained more weight than control mice fed by both standard and lipid rich food (38). All these findings together with the negative association between BMI and serum ZAG levels suggest that ZAG is closely linked to obesity, not only in simple overweight/obese patients, but also in PCAD patients.

Additionally, our present studies found that serum ZAG levels tended to be higher in males than females, and sex was an independent factor associated with serum ZAG levels after adjusting for age, BMI, and other anthropometric and biochemical items. In support of the present results, our previous studies also found that serum ZAG levels of males tended to be higher than those of females in both controls and hypertension patients (18). Similarity, studies performed by Yeung et al. in southern Chinese people and Selva et al. in Caucasian population reported that serum ZAG levels were significantly higher in males (12) and were independently associated with male on multivariate analysis (12, 17). Thus, it is reasonable to analyze the changes serum ZAG levels in terms of gender in order to exclude the effect of gender in the studies. It is well-documented that male is an obvious risk factor of PCAD (25). Our present results showed that subjects with the low tertile ZAG levels were more likely to have PCAD, especially for males. That means though serum ZAG levels were higher in males than in females, the decreased serum ZAG levels in males made them more susceptible to PCAD.

Finally, we found that Cr was significantly higher both in PCAD and NPCAD groups in comparison with controls although it was still within the normal range. Moreover, Cr was an independent factor associated with serum ZAG levels. Up to now, only one literature has reported the positive correlation between serum ZAG and Cr levels in type 2 diabetes mellitus patients in Chinese Han population (39). Our previous studies in type 2 diabetes mellitus showed that patients with the high tertile ZAG levels were more likely to have mildly estimated glomerular filtration rate (eGFR) decrease, which suggested that ZAG might be a potential biomarker for early diagnosis of diabetic nephropathy in patients with T2DM (29). However, the detailed relationship between ZAG and Cr in PCAD patients remains to be illustrated in the future studies.

In summary, serum ZAG levels were firstly found to be decreased in Chinese PCAD patients. PCAD was found to be independently and negatively associated with serum ZAG levels. Subjects with lower ZAG levels were more likely to have PCAD, especially for male subjects with BMI < 24 kg/m^2^. These findings were still remained when diabetic patients in PCAD and NPCAD groups were removed. ZAG might be the potential diagnostic biomarkers for PCAD patients and the combination of ZAG and clinical variables could yield superior discriminative performance. However, our findings were conducted in small samples of Chinese people, and thus may not generalized to other populations. Additionally, as a cross-sectional study, no causal relationship between serum ZAG and PCAD could be drawn in our present studies. The further prospective cohort study and the larger samples study need to be done in the future.

## Author Contributions

ML analyzed the data and wrote the primary manuscript. HZ designed the experiments and revised the primary manuscript. TZ, HP, and YZ collected the clinical materials. LW, HY, and KY collected the serum samples and finished the clinical and biochemical parameters measurements. FG designed the experiment, supervised the whole study, and revised the primary manuscript.

### Conflict of Interest Statement

The authors declare that the research was conducted in the absence of any commercial or financial relationships that could be construed as a potential conflict of interest.

## Abbreviations

ZAG: zinc-α2-glycoprotein
PCAD: premature coronary artery disease
NPCAD: non-premature coronary artery disease
ELISA: enzyme-linked immunosorbent assay
ROC: receiver operating curve
CAD: coronary artery disease
WAT: white adipose tissue
TNF-α: tumor necrosis factor-alpha
AUC: area under the curve
PUMCH: peking union medical college hospital
SBP: systolic blood pressure
DBP: diastolic blood pressure
TC: total cholesterol
TG: triglycerides
LDL-C: low-density lipoprotein cholesterol
HDL-C: high-density lipoprotein cholesterol
FBG: fasting blood glucose
SD: standard deviation
OR: odds ratio
CI: confidence interval.
