# Supplementary material for: Serum Zinc-α2-Glycoprotein Levels Were Decreased in Patients With Premature Coronary Artery Disease
Source: Front Endocrinol (Lausanne). 2019 Mar 29;10:197. doi: 10.3389/fendo.2019.00197 (PMC6449697; doi:10.3389/fendo.2019.00197)
Supplement: Supplementary file 1 [file Table_1.DOCX]

**Table S1. Unconditional logistic regression analysis of PCAD/NPCAD risks according to the tertiles of serum ZAG when diabetic patients were removed.**

|  | **Tertile** | | |
| --- | --- | --- | --- |
| **Measurement** | **Lowest**  **OR (95% CI)** | **Median**  **OR (95% CI)** | **Highest**  **OR (95% CI)** |
| **ZAG in PCAD vs. Control** | | | |
| Range (μg/mL)  Cases/controls | <7.809  104/37 | ≧7.809 to <8.676  76/65 | ≧8.676  62/80 |
| Model 1 | 2.657 (1.261-5.595) | 1.527 (0.739-3.157) | 1.00 (reference) |
| *p* | **0.010** | 0.253 |  |
| Model 2 | 3.006 (1.246-7.253) | 1.632 (0.689-3.866) | 1.00 (reference) |
| *p* | **0.014** | 0.265 |  |
| Model 3 | 3.648 (1.432-9.292) | 2.252 (0.885-5.732) | 1.00 (reference) |
| *p* | **0.007** | 0.089 |  |
| **ZAG in NPCAD vs. Control** | | | |
| Range (μg/mL) | <8.041 | ≧8.041 to <8.944 | ≧8.944 |
| Cases/controls | 44/49 | 22/70 | 31/63 |
| Model 1 | 1.886 (0.859-4.139) | 1.00 (reference) | 1.972 (0.846-4.599) |
| *p* | 0.114 |  | 0.116 |
| Model 2 | 2.177 (0.828-5.720) | 1.00 (reference) | 2.332 (0.869-6.254) |
| *p* | 0.115 |  | 0.093 |
| Model 3 | 2.302 (0.803-6.598) | 1.00 (reference) | 1.912 (0.641-5.707) |
| *p* | 0.121 |  | 0.245 |
| **ZAG in PCAD vs. NPCAD** | | | |
| Range (μg/mL) | <7.564 | ≧7.564 to <8.549 | ≧8.549 |
| Cases/controls | 86/27 | 81/32 | 75/38 |
| Model 1 | 1.655 (0.871-3.143) | 1.522 (0.820-2.826) | 1.00 (reference) |
| *p* | 0.124 | 0.184 |  |
| Model 2 | 1.480 (0.733-2.988) | 1.257 (0.629-2.512) | 1.00 (reference) |
| *p* | 0.275 | 0.518 |  |
| Model 3 | 1.452 (0.705-2.989) | 1.150 (0.563-2.349) | 1.00 (reference) |
| *p* | 0.312 | 0.701 |  |

Multivariate odds ratios (OR) and 95% confident intervals (CI) from unconditional logistic regression models were used in the analysis.

Model 1: basic model, adjusted for age, gender, BMI (<24 kg/m^2^, ≥24 kg/m^2^), SBP and ln-FBG.

Model 2: further adjusted for ln-TC and ln-HDL-C based on the model 1.

Model 3: Full model, further adjusted for ln-Cr and ln-Urea based on the model 2.
