# Supplementary figures and images for: Serum Zinc-α2-Glycoprotein Levels Were Decreased in Patients With Premature Coronary Artery Disease
Source: Front Endocrinol (Lausanne). 2019 Mar 29;10:197. doi: 10.3389/fendo.2019.00197 (PMC6449697; doi:10.3389/fendo.2019.00197)

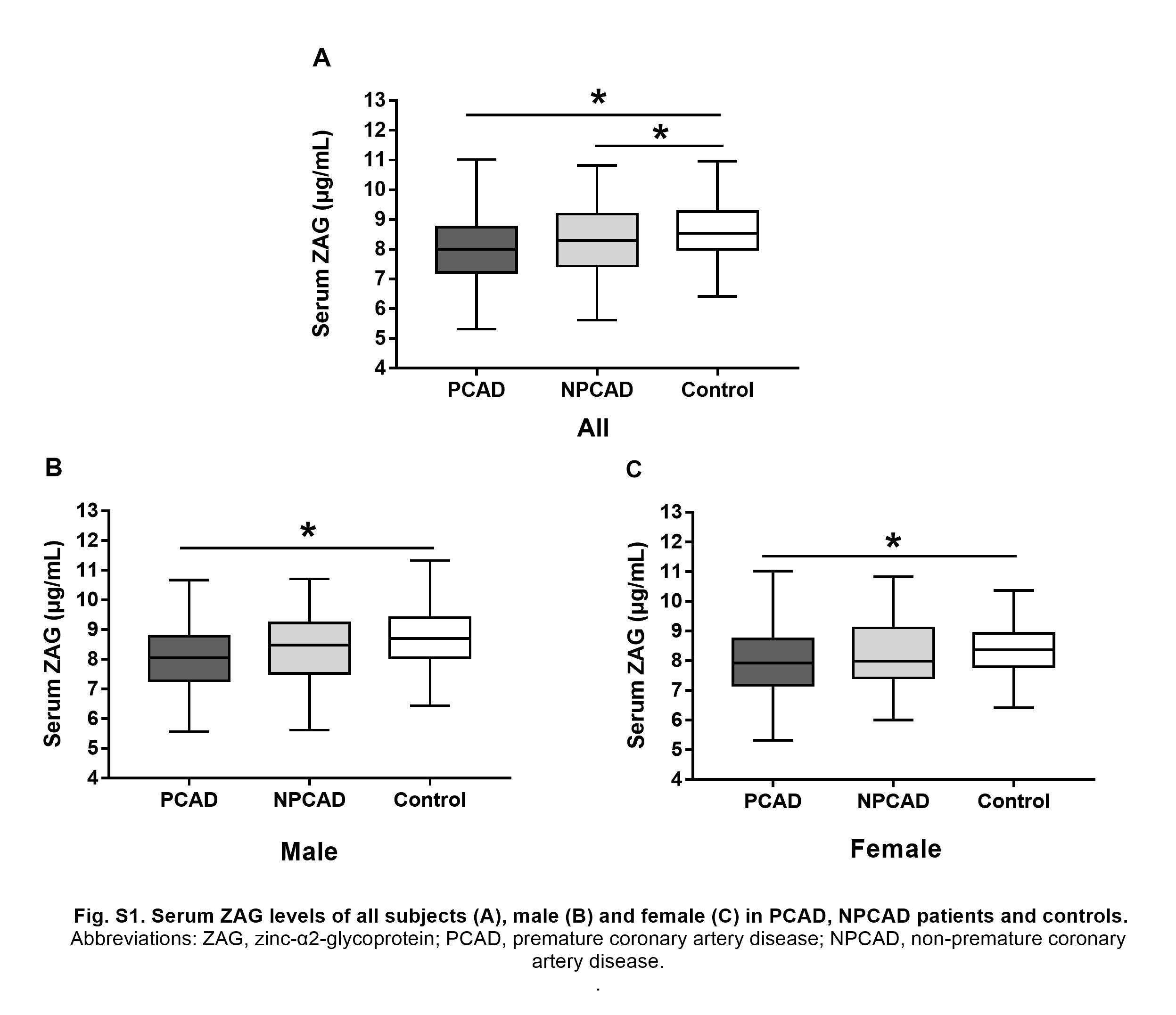

Supplement: Supplementary file 2 [file Image_1.JPEG]

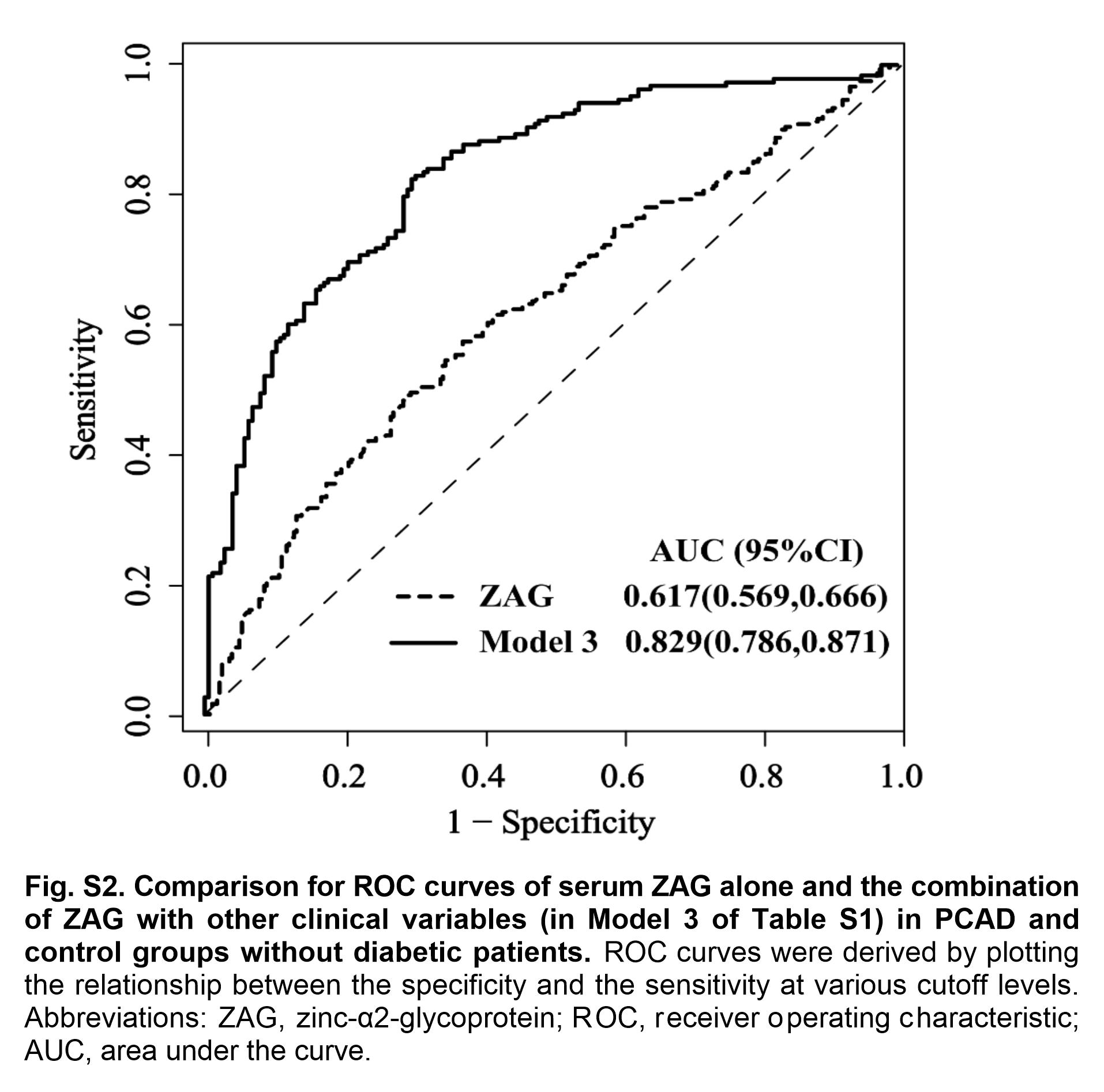

Supplement: Supplementary file 3 [file Image_2.JPEG]
